# Vernalization or devernalization? A question about *VRT2*

**DOI:** 10.1093/plphys/kiae440

**Published:** 2024-08-22

**Authors:** Dechang Cao

**Affiliations:** Assistant Features Editor, Plant Physiology, American Society of Plant Biologists; Germplasm Bank of Wild Species & Yunnan Key Laboratory for Crop Wild Relatives Omics, Kunming Institute of Botany, Chinese Academy of Sciences, Kunming, Yunnan 650201, China

As the Earth revolves, the seasons change. Organisms perceive these changes in their own ways to enhance their fitness. As wise as human beings, plants can distinguish spring and autumn despite similar temperatures. Vernalization takes the center stage in plants' perception of spring versus autumn. Vernalization is a programmed physiological process in which prolonged exposure to chilling promotes flowering (Xu and Chong 2018). Vernalized plants are prompted to flower in spring, while the absence of vernalization in summer decreases the risk of flowering in autumn and being damaged in winter.

**Figure 1.**
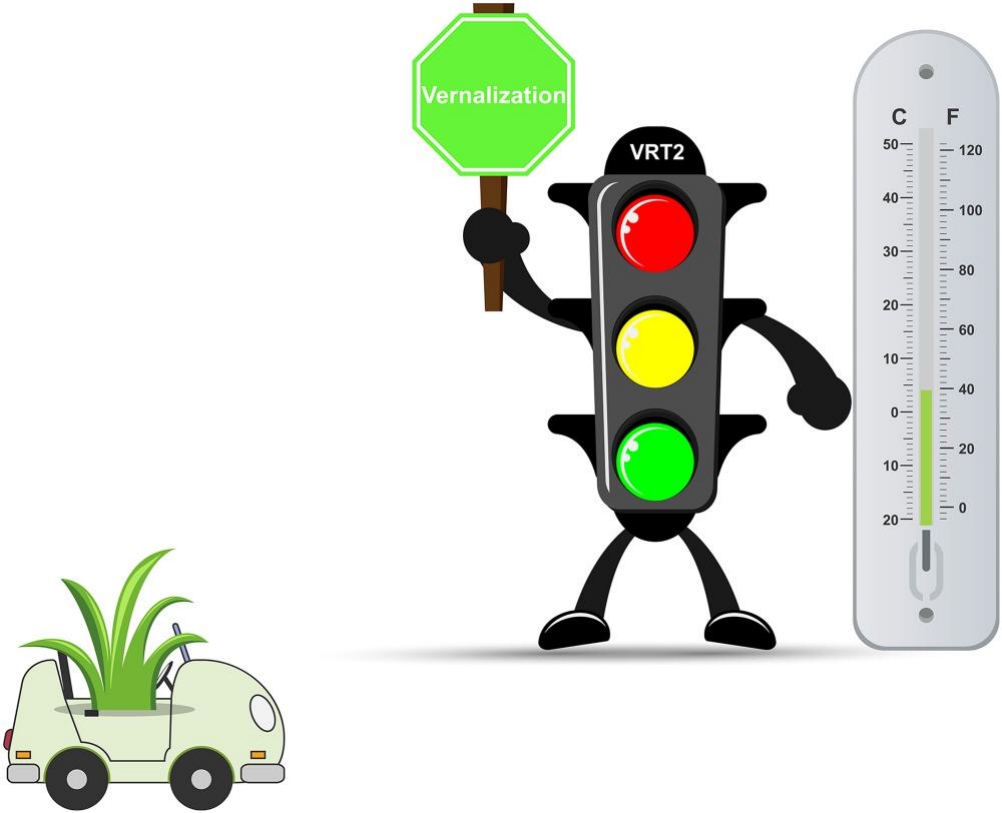
*VRT2* plays an important role in regulating vernalization, stabilization of vernalization, and devernalization in a temperature-dependent manner. Bd*VRT2* exhibited dramatically increased expression during vernalization in *Brachypodium distachyon*. Compared with wild-type plants, loss-of-function mutants of *BdVRT2* showed prolonged requirements for saturated vernalization and increased sensitivity to warm temperature-induced devernalization. Moderate temperature is helpful to stabilize vernalization of *B. distachyon*, and *BdVRT2* is required to fulfill the stabilization. The cartoons were adapted from free vectors on www.vecteezy.com.

As a consequence of global climate change, we have more extreme and unpredictable weather. It is an urgent need to increase the capability of plants to cope with fluctuating and variable warm winters. Knowledge about vernalization and its stabilization is crucial for crop production. While some plants overwinter in the form of seedlings and receive bud vernalization, seed vernalization that breaks dormancy also accelerates flowering (Penfield and Springthorpe 2012). Some pleiotropic genes may be involved in both seed dormancy and flowering, thus linking seed vernalization and early flowering (Auge et al. 2019). Some epigenetic mechanisms also are responsible for vernalization to help plants remember winter (Xu and Chong 2018). In this issue of *Plant Physiology*, Kennedy et al. (2024) reported temperature-dependent vernalization and devernalization responses mediated by *VEGETATIVE TO REPRODUCTIVE TRANSITION 2* (*VRT2*) in a temperate grass *Brachypodium distachyon* (Poaceae) (Fig. 1).

*B. distachyon* (accession Bd21-3) required 3 months to start flowering when planted at 22 °C in long-day conditions (16-h photoperiod), and seed vernalization at 4 °C for 2 weeks before sowing substantially accelerated flowering time by 2 months. Interestingly, a heat break at 32 °C (for 1 week) after 2 weeks of seed vernalization substantially delayed the flowering, suggesting that the vernalization effect could be revoked (devernalization). Devernalization was also observed in other plants (Preston and Fjellheim 2022). However, when vernalization was extended to 4 weeks, flowering of the plants was no longer reversed by an exposure to 32 °C. Thus, it seems that a long saturated vernalization signifies a convincing winter, while a short partial one does not.

Further, Kennedy et al. (2024) found that the effect of vernalization can be stabilized in *B. distachyon* by medium temperature. While partial 2-week vernalization of *B. distachyon* was reversed by high temperature of 32 °C (resulting in delayed flowering), an incubation of the partially vernalized seeds at 18 °C for 1 week before exposure to 32 °C led to an early flowering, thus stabilizing vernalization. Exposure to medium temperatures also stabilizes vernalization in Arabidopsis and chicory (Preston and Fjellheim 2022).

Epigenetic regulation of *FLOWERING LOCUS C* (*FLC*) was shown to stabilize vernalization in Arabidopsis (Penfield et al. 2021). The floral repressor *FLC* was initially H3K27me3-methylated on the first intron in response to vernalization. Subsequently, further vernalization spreads H3K27me3 methylation across the *FLC* locus and leads to silencing and stabilization of vernalization. However, vernalization responses evolved independently among plants (Larran et al. 2023), and it remains largely unknown how vernalization is maintained and reversed in the model grass *B. distachyon*.

*VRT2*, a gene encoding a Short Vegetative Phase (SVP)-like MADS-box transcriptional factor, recently came to the spotlight of vernalization in grass species. *VRT2* showed vernalization-dependent expression in wheat (Xie et al. 2021), and the H3K27 methylation level of *VRT2* increased during vernalization in *B. distachyon* (Huan et al. 2018). Time-course gene expression of *BdVRT2* was investigated during vernalization to estimate the role of *VRT2* in vernalization responses of *B. distachyon*. When the *B. distachyon* plants were grown at ambient temperatures (in an unheated greenhouse), the mRNA abundance of *BdVRT2* increased by around 20-fold in winter and maintained a high level after reaching a peak. However, this vernalization-dependent expression pattern was not observed when the plants were vernalized under strictly controlled conditions of 4 °C (8-h photoperiod) during 4 weeks. This difference may be attributed to the temperature fluctuation and gradual changes in photoperiod in the unheated greenhouse.

To further characterize the role of *VRT2* in vernalization responses of *B. distachyon*, loss-of-function *bdvrt2* T-DNA mutants were examined. T-DNA mutants of *bdvrt2* showed delayed flowering compared with control plants when they were grown at 18 °C after vernalization at 4 °C for 2 weeks, suggesting a substantial role of *BdVRT2* in vernalization. More interestingly, the delay in flowering was dependent on temperature, with a prolonged delay observed when plants were grown at 24 °C post vernalization. The temperature-dependent delay in flowering of *bdvrt2* is in accordance with the observation that 1-week vernalized *B. distachyon* plants showed differential flowering time at 18 °C (earlier) and 24 °C (delayed). These results suggested that *BdVRT2* was also involved in devernalization in *B. distachyon*. Knockout mutants of *BdVRT2* generated using CRISPR validated that *BdVRT2*-mediated vernalization response was sensitive to temperature. Thus, vernalization and devernalization are likely integrated by *BdVRT2* in *B. distachyon*.

When the authors extended seed vernalization to 4 weeks, it was found that the extreme delay in flowering of *bdvrt2* was alleviated, suggesting a saturated vernalization was achieved after a longer period of vernalization than the null sibling control plants. Observations in CRISPR knockout mutants of *BdVRT2* showed an even longer period to saturate vernalization (8 weeks). Thus, *BdVRT2* is an important determinant of the time requirement for full vernalization in *B. distachyon*.

To further estimate the role of *BdVRT2* in stabilization of vernalization, the authors disrupted the vernalization process of *bdvrt2* and wild-type seeds to compare their flowering phenotypes. Seeds vernalized at 4 °C for 1 week were moved to 18 °C for various days and returned to 4 °C to achieve a total of 4 weeks vernalization for all treatments. The wild-type plants showed relatively stable flowering time, while flowering of the *bdvrt2* mutant was notably delayed when vernalization was disrupted for 4 days. These results suggest that *BdVRT2* plays an important role in stabilizing and restarting vernalization after interruption.

A comprehensive comparison of gene expression was conducted between *bdvrt2* and null sibling control plants with different status of vernalization. RNAseq revealed 254, 80, and 155 differentially expressed genes when plants were partially vernalized (4 °C for 3 days), stabilized (18 °C after 2-weeks vernalization), and devernalized (24 °C after 2-weeks vernalization), respectively. It was interesting that 14 DEGs were shared among the 3 sets of RNAseq data. One shared gene was *BdSUZ12*, which is a Polycomb Repressive Complex 2 (PRC2) gene, and its homolog, *EMBRYONIC FLOWER2* (*EMF2*), in *Arabidopsis* is a known epigenetic repressor of floral transition (Yoshida et al. 2001). *BdSUZ12* showed higher expression levels in the *bdvrt2* mutants than the null sibling control plants at all conditions, suggesting *BdVRT2* might play a role in repressing the epigenetic regulators of vernalization. Taken together, the findings have demonstrated that *BdVRT2* is an important determinant of temperature-sensitive vernalization and devernalization responses in the grass *B. distachyon*, providing useful clues for our crop improvement strategies to cope with more and more variable winter weather in the future.
